# Comparison of Food Intake in Multiple Sclerosis Patients and Healthy Individuals: A Hospital-Based Case-Controlled Study

**Published:** 2019

**Authors:** Yasaman GHAZAVI, Zahra BAHADORAN, Mana NIKFARJAM, Nahid Beladi MOGHADDAM, Parvin MIRMIRAN, Mohsen Reza HEYDARI

**Affiliations:** 1 Faculty of Medicine, Shahid Beheshti University of Medical Sciences, Tehran, Iran; 2 Nutrition and Endocrine Research Institute, Shahid Beheshti University of Medical Sciences, Tehran, Iran; 3 Department of Neurology, Faculty of Medicine, Shahid Beheshti University of Medical Sciences, Tehran, Iran; 4 Department of Neurology, Faculty of Medicine, Baqiyatallah University of Medical Sciences, Tehran, Iran

**Keywords:** Multiple sclerosis, Diet, Food frequency questionnaire, Nutrition, Meat, Case-control study

## Abstract

**Objectives:**

Nutritional factors affect the incidence, severity of symptoms and progression of multiple sclerosis (MS). However, the role of specific nutritional factors remains largely unknown in MS. We conducted this hospital-based case-controlled study to investigate the association between dietary intake and risk of MS.

**Materials & Methods:**

This study was conducted on 93 MS patients and 94 age-matched controls from Oct 2015 to Sep 2016 in Tehran, Iran. MS was diagnosed based on 2010 McDonald criteria and Brain Magnetic Resonance Imaging. Dietary intake was assessed using a validated semi-quantitative food frequency questionnaire. Odds ratio and 95% confidence interval of MS was calculated in different food groups using multiple logistic regression models adjusted for potentially confounding variables and compared between the two groups.

**Results:**

There was no significant difference between the age (34.62 ±9.68 vs. 33.96±8.75) and BMI (23.96 ±4.07 vs. 24.47 ±4.07) of MS and control group, respectively. Higher intake of processed meat (OR (95% CI))=(2.07(1.18-3.63) and non-processed meat (1.38(1.13-1.68)) were found in the MS group compared with the control.

**Conclusion:**

Higher intake of processed meat and non-processed meat was associated with increased risk of MS. Further studies on the probable role of these nutritional factors in the pathogenesis of MS are suggested.

## Introduction

Multiple sclerosis (MS) is a neurodegenerative disease of the central nervous system, with an autoimmune inflammatory process leading to blood-brain barrier disruption, perivascular inflammation, demyelination, axonal damage, and progressive neuronal loss. Precise etiology of MS is not clearly identified yet, though immune dysregulation due to genetic susceptibility and multiple environmental factors might have a role (1, 2). Inflammatory and neurodegenerative processes are the two factors defining the course of the disease and the clinical phenotype. MS has been classified into four clinical subtypes: relapsing-remitting, progressive relapsing, primary progressive and secondary progressive. Lately, this classic description has been redefined in accordance with the disease activity and the clinical progression of the disease (2). The neurodegenerative disease disables patients with a wide range of chronic symptoms including impairment in mobility, sensory function, vision, cognition, bowel/bladder function, spasticity, pain, fatigue, depression, and tremor/coordination domains (3). Genetics, altered immune system and other environmental factors (infection and nutrition) might have a role on etiology of MS, though the exact etiology of the disease is still unknown (4). There is a geographical variation in MS incidence shown that risk of MS varies with migration in childhood, emphasizing on environmental factors as a risk factor for MS. 

Nutritional factors have an effect on incidence and severity of MS. However, the exact role of specific nutritional factors is not known yet (5). Higher intake of vitamin D has been correlated with decreased incidence of MS as shown in epidemiological studies (6). A considerable number of controlled clinical trials have failed to be conclusive on the role of diet in MS mainly due to limitations of study design, patient characteristics and the number of samples (7). 

There is an increasing incidence of MS in Tehran, Iran, and the prevalence was reported as high as 74 in 100,000 in 2013 (8). “Lifetime use of dietary intervention was reported by 41% of people with MS in a study from Germany” (9). At present, no dietary guidelines are available for MS patients and individuals at risk. Associated studies have been focusing on the role of vitamin D and obesity that are both diet-dependent (10, 11).

There are considerable number of MS patients using dietary interventions and many diet-related comorbidities such as malnutrition, metabolic syndrome, and cardiovascular disease, seem to diminish the quality of life in MS patients. Thus, there is a need to evaluate the role of diet in MS with evidence-based methods in order to further provide an appropriate nutritional counselling to MS patients (12).

In a current case-control study, we examined the association between consumption of different food-group intake and the risk of MS and compared the macro and micro-nutrient intake between MS patients and healthy controls in Tehran

## Materials & Methods

***Participants and Study design***

Participants were enrolled in this case-control study of 93 consecutive patients with MS and 94 healthy subjects, group matched for age (years) and sex (male/female) in Tehran, Iran. Participants were recruited among patients aged 18-56 yr referred to Imam Hossein Hospital, Tehran, Iran from Oct 2015 to Sep 2016. MS was diagnosed by neurologist based on 2010 McDonald criteria and Brain Magnetic resonance imaging (13, 14). The control group was formed by 94 sex- and age-matched healthy individuals accompanying the patients in same hospital. 

**Figure 1:**
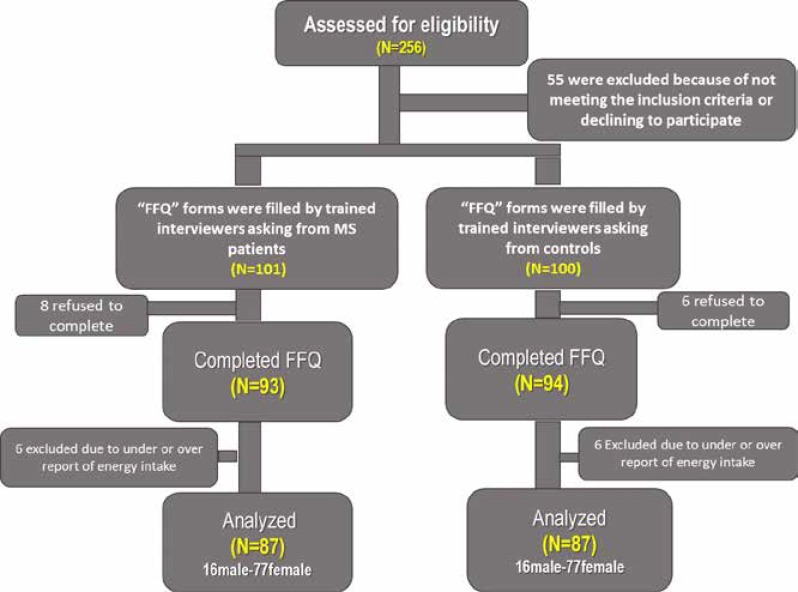
Study fellow chart

Our inclusion criteria were an age limit of 18 to 70 yr, MS diagnosed based on 2010 McDonald criteria and Brain MRI, having no diet modification since the start of course of the disease, having no other systemic disease, nonsmoker, taking no additional supplements before starting the course of the disease. Patients leaving more than 10 food items of questionnaire blank or having a total energy intake less than 800 Kcal or more than 4200 Kcal were excluded (15). The final study sample included 87 cases and 87 controls after applying the exclusion criteria (Figure 1). 

The study was approved by the medical research Ethics Committee of Shahid Beheshti University of Medical Sciences. Written informed consent was obtained from all study participants. 

***Assessment of dietary intake***

We interviewed all the MS patients and control subjects using structured pretested questionnaires. The demographic characteristics (age (years), sex (male/female), BMI (kg/m2)), disease duration, Vitamin D supplementation before the onset of disease, family history of MS and the climate zone of the city they lived in the first 15 years of their life based on world map of Koppen-Geiger for climate in a demographic questionnaire dietary intake were noted (16). The FFQ contained a checklist of food items with a standard serving size regularly used by Iranians. Reliability of the questionnaire was confirmed by a previous study reported by Shahid Beheshti Endocrine Research Institute (17). Participants were asked to address their frequency of consumption of a given serving of each food item during the previous year on a daily, weekly or monthly assumption. The stated frequency of consumption of food items was then converted to daily intake. While using household measures, portion sizes of consumed foods were converted to grams. Nutrient consumption was then calculated using the Nutrients Composition of Iranian Foods Supplemented with the United States Department of Agriculture (USDA) Food Composition Data (18).

***Statistical methods***

The SPSS software ver. 18 (SPSS Inc., Chicago, IL, USA) was used for all statistical analyses. Normality of the distributions of dietary intake variables was assessed by the Kolmogorov-Smirnov test. Means and standard deviations were calculated for energy and all food items and nutrient intakes from FFQ. Energy-adjusted nutrient intakes were calculated to remove variation due to energy, using the residual method (19). For data with normal distribution the independent sample t-test and for data not normally distributed, the Mann-Whitney test was conducted to compare differences between the mean dietary intake of micronutrients between the two groups of MS patients and healthy controls. We also divided food items into 13 food groups including grains, total dairy, legumes, meat, processed meat, total vegetables, starchy vegetables, fruits, nuts, sugars, oil, salty snacks and sweet snacks. The food groups were then analyzed both as a continuous variable and as a categorical variable. Odds Ratio and 95% Confidence interval for the risk of MS were estimated using logistic regression models adjusted for age, sex, BMI, family history of MS, Vitamin D supplementation before the onset of disease and the temperature zone they lived in the first 15 years of their life, total energy intake, total fiber and total fat.

## Results

This study was conducted on 93 MS patients and 94 in the control group. The mean age of participants was mean ±SD =34.6±9.6 yr in MS and 34.9±8.7 yr in controls and their mean BMI was 23.4±4.0 kg/m2 in MS patients and 24.4 ±3.8 kg/m2 in controls. Mean total calorie intake was 2631+724 kcal in MS and 2683±680 kcal in controls. Percent of male gender was 16% in MS patients and 19% in controls. The family history of MS was higher among MS patients (12%) than the controls (5%) (Table 1). There was no significant difference in intake of total energy, total carbohydrate, total protein and total fat between MS and control group but the MS patients had a significantly lower intake of total fibers (*P*=0.024) (Table 2). There was a significantly lower intake of sodium (7820±1274 mg/day vs 8395±1592 mg/day) (*P*=0.03) and lycopene (6310± 2651 µg/day vs. 7615± 2712 µg/day) (*P*=0.02) in MS patients compared to the control group. 

Mean intake of food groups and OR and 95% CI for the risk of MS are shown in Tables 3 and 4. After adjustment for potential confounding variables, results obtained from modeling food groups as a continuous variable in relation to the risk of MS showed that the intake of processed meat (OR=2.07; 95% CI=1.18-3.63), and non-processed meat (1.38; 1.13-1.68), increased the risk of MS. In this study, processed meat group was consisted of packaged tuna fish, sausage, hamburger and ham. Non-processed meat group consisted of beef, lamb, veal, chicken and fish meat. No significant difference between the intake of other food groups and risk of MS was observed. Although the *P*-value of sweet snacks and starchy vegetables were significant (Table 3), the OR and CI did not confirm a positive correlation (Table 4).

**Table 1:** Characteristics of cases and controls

Characteristics		MS	Control	*P*-value
		(n= 93 )	(n= 94)	
Age (years, mean ± SD)		34.62± 9.8	34.96± 8.75	0.64
Male (percent)		16%	19%	_
BMI (Kg/m^2^ mean± SD)		23.46± 4.07	24.47± 3.81	0.12
Disease duration (years)		7.34± 6.1	_	_
Positive family history of MS (percent)		12%	5%	_
Climate classification of location* (percent)				
Arid(Dessert-hot arid)		3%	1%	_
Warm temperate(steppe-hot summer/dessert-warm summer)		11%	41%	_
Snow(Steppe-warm summer)		85%	57%	_
Vitamin D supplementation before disease onset		0.05%	0%	_
MS subtype:				
Relapsing-remitting ms		40%	_	_
Progressive relapsing ms		17.50%	_	_
Clinically isolated syndrome		12.50%	_	_
Secondary progressive ms		7.50%	_	_

Climate of the city they lived in first 15 years of life based on World Map of the Köppen-Geiger climate

classification. The percentage of MS cases living in lower temperature zone (steppe warm -summer)

was higher than control group (85% vs 57%). The MS subtype of participants Included: 40% Relapsing-

Remitting, 17.50% Progressive Relapsing, 12.50% Clinically Isolated Syndrome and 7.50% Secondary

Progressive MSclassification

**Table 2:** Dietary intake of the study participants*

	MS"	Control"	*P*-value
Energy (Kcal)	2631±724	2683±680	0.31
Carbohydrate(g)	772±116	762± 99	0.55
Total fat(g)	191±31.9	199±31.1	0.14
Protein(g)	175±27.2	179±26.4	0.24
Total fiber(g)	90±20	93±21	0.024

Data are mean ± standard deviation

*Mean daily intake of energy, macronutrients and fiber were compared between MS and control using independent sample T-test

**Table 3:** Dietary intake of the study participants*

	MS	Control	*P-*value
Grains	534.51±371.25	508.87±179.11	0.31
Total Dairy	499.44±435.49	355.93±231.93	0.19
Legumes	51.98±69.84	50.28±55.04	0.42
Meat (non-processed)	67.2±78.32	77.62±52.44	0.01
Processed Meat	9.66±12.86	16.38±22.76	0.01
Total Vegetables	341.95±257.19	400.28±243.81	0.14
Starchy vegetables	37.38±27.08	43.33±31.98	0.009
Fruits	491.18±369.27	474.55±371.86	0.88
Nuts	29.93±127.14	15.14±21.44	0.65
Sugars	152.55±23.24	151.73±23.24	0.27
Oils	36.42±31.13	40.45±43.71	0.11
Salty snacks	21.11±27.86	17.98±17.80	0.67
Sweet snacks	43.19±51.30	30.61±25.52	0.03

*Mean daily intake of food groups (grams/day) ± Standard deviation

**Table 4:** Odds ratio and 95% confidence interval for the association of food group intake and multiple sclerosis *

Tertile categories of dietary intake(g/day)				Dietary intake(g/day)		
	OR (95% CI)			OR(95% CI)		
	T1	T2	T3			P-value
Grains	†70.7	81.7	98.7			
Model1 ‡	1	1.03(0.51-2.07)	1.00(0.49-2.01)	0.99(0.98-1.00)		0.54
Model2 §	1	0.75(0.23-2.39)	0.75(0.23-2.43)	0.98(0.94-1.01)		0.27
Total Dairy	27.8	43.6	67.6			
Model1	1	1.03(0.51-2.07)	1.00(0.49-2.01)	0.98(0.97-0.99)		0.009
Model2	1	0.69(0.23-2.02)	2.05(0.61-6.81)	0.98(0.96-1.01)		0.3
Legumes	4.4	5.9	9.8			
Model1	1	1.43(0.33-6.14)	1.34(0.45-4.03)	0.85(0.95-1.04)		0.99
Model2	1	0.74(0.23-2.36)	0.74(0.23-2.39)	0.99(0.91-1.09)		0.97
Meat (non-processed)	7.03	9.68	13.1			
Model1	1	1.64(0.44-6.19)	2.35(0.54-10.34)	1.02(0.97-1.07)		0.28
Model2	1	1.23(0.44-3.47)	1.49(0.39-5.59)	1.38(1.13-1.68)		0.001
Processed Meat	0.449	0.913	2.11			
Model1	1	1.11(0.23-3.87)	2.12(0.45-10.3)	1.25(1.03-1.51)		0.021
Model2	1	1.24(0.43-4.63)	1.42(0.43-4.63)	2.07(1.18-3.63)		0.01
Total Vegetables	38.3	52.2	75.1			
Model1	1	0.78(0.36-2.94)	0.84(0.21-3.34)	1.01(0.99-1.02)		0.11
Model2	1	1.22(0.42-3.51)	1.33(0.36-4.92)	1.04(1.00-1.07)		0.01
Starchy vegetables	4.2	5.9	7.8			
Model1	1	2.43(0.56-10.38)	3.34(0.83-11.65)	1.07(0.99-1.18)		0.12
Model2	1	1.83(0.59-5.64)	1.08(0.33-3.49)	1.17(0.97-1.41)		0.88
Fruits	45.5	56.9	83.1			
Model1	1	1.33(0.31-3.23)	0.94(0.26-3.34)	0.99(0.99-1.00)		0.75
Model2	1	1.39(0.45-4.30)	1.00(0.21-4.43)	1(0.98_1.02)		0.66
Nuts	0.11	0.99	2.09			
Model1	1	1.12(0.33-4.23)	1.04(0.33-3.85)	0.93(0.90-1.04)		0.39
Model2	1	1.74(0.59-5.10)	1.04(0.30-3.61)	0.95(0.72-1.25)		0.72
Sugars	3.4	5.4	12.5			
Model1	1	0.83(0.18-2.54)	3.65(0.54-16.23)	0.98(0.86-1.11)		0.8
Model2	1	0.54(0.16-1.82)	0.78(0.22-2.67)	1.01(0.81-1.26)		0.9
Oils	3.9	5.4	7.7			
Model1	1	0.73(0.10-3.32)	0.63(0.15-3.66)	1.03(0.95-1.11)		0.47
Model2	1	0.26(0.07-0.96)	0.09(0.01-5)	0.75(0.6-0.93)		0.01
Salty snacks	1.2	1.9	3.3			
Model1	1	1.32(0.32-4.89)	1.72(0.23-5.53)	0.94(0.82_1.07)		0.36
Model2	1	0.71(0.23-2.20)	0.63(0.17-2.23)	0.89(0.69-1.15)		0.38
Sweet snacks	2.1	3.6	6.5			
Model1	1	0.84(0.24-4.23)	3.23(0.66-9.12)	0.91(0.84_0.99)		0.04
Model2	1	1.46(0.46-4.65)	4.76(1.22-18.61)	1.03(0.86-1.24)		0.71

* Multivariable logistic regression models were used with adjustment of potential confounders.

† Median of intake of food group in each tertile category, used as a continuous variable in logistic regression models(10grams/day)

‡ Adjusted for energy(Kcal)

§ Additional adjustment for Body mass index(BMI) (kg/m2), sex(male, female), family history of multiple sclerosis(yes, no) vitamin D supplementation before onset of disease(yes, no), city they lived in the first 15 years of life(arid, humid, snow), total energy (kcal), total fibers(grams) and total fat (grams)

## Discussion

In this study, intake of different food groups and nutrients in MS patients and controls were evaluated. Our results suggest that the consumption of different kinds of meat is associated with increased risk of MS, supported by several epidemiological studies (20, 21). Other case-control studies have similarly illustrated that consumption of meat is positively associated with MS (22-25). In contrast, a case-control study of 226 women in Ahvaz, Iran revealed no association between the total amount of consumed meat and the risk of MS (26). However, the limited number of questions in a self-made questionnaire with no previous reliability check was perhaps inappropriate in this study.

Several mechanisms have been proposed to explain the association between meat intake and MS risk. Firstly, red meat is a source of N-glycolylneuraminic acid (Neu5Gc), which is a sialic acid not originally made in the human body. This molecule found in meat could be related to chronic inflammation as there are anti Neu5Gc antibodies found in the human body (27). Secondly, the effect of meat can be related to saturated animal fat it contains. Saturated fats can lead to obstruction of small capillaries. Moreover, the effect of fat on the cells occurs at the level of gene expression and cell growth and differentiation, which could be the cause of chronic inflammation (28, 29). Finally, the role of meat in increasing the risk of MS could be due to consumption of amines and nitrosoamines in red meat (especially processed meat) that facilitate the formation of endogenous nitroso-compounds (NOCs). Formation of NOCs in the body is directly related to the amount of meat consumed. NOCs are mutagenic and could cause damage to DNA, thus consumption of processed meat can increase MS risk (30).

Role of diet and lifestyle on MS is still controversial and they are not considered as a part of the therapeutic approach in MS patients. However, nutritional factors and lifestyle modification might have an alleviating or aggregating role in MS, caused by the metabolic and inflammatory pathways inside cells. Specific diet can drive cells to produce pro-inflammatory molecules. The western high-calorie diet consisting of high salt, high fat, red meat, sugar-sweetened beverages, fried food, low fiber and insufficient physical activity are the factors leading to inflammation (4). Consumption of different food groups in western diet consisted of meat, processed meat, sweets and deserts has shown a positive correlation with inflammatory markers in serum (31). In addition, consumption of foods with higher inflammatory index (proinflammatory) was associated with increased risk of MS (32). In accordance with the findings on proinflammatory pathways of foods, proper nutritional intervention has been recommended for MS patients (32).

Lower intake of lycopene in MS patients compared to the control group was found in this study that warrants broader studies to characterize a temporal relationship. Lycopene is a carotenoid found in tomato, carrot, watermelon and some other fruits and vegetables with antioxidant and anti-inflammatory effects (25). Neurons are sensitive to oxidative stress due to high consumption of oxygen as the neuronal membrane contains high amounts of polyunsaturated fatty acids that do not have a strong anti-oxidant effect (33). As oxygen radicals are observed in high amounts in the brain of people with neurodegenerative diseases, reactive oxygen species (ROS) might have a significant role (34, 35). Although ROS might not be the main factor, they are likely to exacerbate disease progression through the oxidative damage and interaction with mitochondria (35). ROS are chemically reactive molecules naturally generated in biological systems known for having a role in cell survival, stress responses and mediating cellular activity such as inflammation (36). Brain white matter had a lower anti-oxidant activity and thus was more sensitive to damage from free oxygen radicals (24). Hypothetically, significantly lower intake of lycopene in MS patients in our study lead to decreased anti-oxidant and anti-inflammatory effects of lycopene as a protective factor in MS, though further studies are required. In contrast, no associations between intake of dietary carotenoids and risk of MS were reported in a large cohort study (37).

There are several points considered as strengths of our study. To our knowledge, limited studies have examined dietary factors related to MS risk. Evaluating food groups’ intake and micronutrient intake at the same time while comparing them between MS patients and control groups is a unique feature of this study. Our study was carried out in Tehran which is among the cities with the highest rate of MS in Iran (38). Moreover, we were able to conduct the study in a province with a high point prevalence of MS (39). 

Our study has several limitations. Although the inclusion criteria in this study were that patients should not have changed their diet since the diagnosis of the disease, the participants might have not completely reported minor diet alterations due to recall bias. In order to decrease this effect, we used a validated food frequency questionnaire, with which the outcome was compared to several daily recalls in a previous study (17). Although small sample size and case-control sets are considered as weaknesses of the current study, use of a validated semi-quantitative FFQ for dietary assessment and use of several statistical models with adjustment of potential confounding variables were the strengths of this study.

**In conclusion,** a positive association was found between dietary intake of processed and non-processed meat with the risk of multiple sclerosis in the patients of our study.

## References

[1] Hadgkiss EJ, Jelinek GA, Weiland TJ, Pereira NG, Marck CH, van der Meer DM (2015). The association of diet with quality of life, disability, and relapse rate in an international sample of people with multiple sclerosis. Nutr Neurosci.

[2] Lublin FD, Reingold SC, Cohen JA, Cutter GR, Sorensen PS, Thompson AJ (2014). Defining the clinical course of multiple sclerosis: the 2013 revisions. Neurology.

[3] Kister I, Bacon TE, Chamot E, Salter AR, Cutter GR, Kalina JT (2013). Natural history of multiple sclerosis symptoms. Int J MS Care.

[4] Riccio P (2011). The molecular basis of nutritional intervention in multiple sclerosis: a narrative review. Complement Ther Med.

[5] Ascherio A, Munger KL (2007). Environmental risk factors for multiple sclerosis. Part II: Noninfectious factors. Ann Neurol.

[6] Ebers GC (2008). Environmental factors and multiple sclerosis. Lancet Neurol.

[7] Ontaneda D, Thompson AJ, Fox RJ (2017). Cohen Progressive multiple sclerosis: prospects for disease therapy repair and restoration of unction. Lancet.

[8] Etemadifar M, Sajjadi S, Nasr Z, Firoozeei TS, Abtahi SH, Akbari M (2013). Epidemiology of multiple sclerosis in Iran: a systematic review. Eur Neurol.

[9] Schwarz S, Knorr C, Geiger H, Flachenecker P (2008). Complementary and alternative medicine for multiple sclerosis. Mult Scler.

[10] Munger KL, Bentzen J, Laursen B, Stenager E, Koch-Henriksen N, Sorensen TI (2013). Childhood body mass index and multiple sclerosis risk: a long-term cohort study. Mult Scler.

[11] Simon KC, Munger KL, Ascherio A (2012). Vitamin D and multiple sclerosis: epidemiology, immunology, and genetics. Curr Opin Neurol.

[12] Esposito S, Bonavita S, Sparaco M, Gallo A, Tedeschi G (2017). The role of diet in multiple sclerosis: A review. Nutr Neurosci.

[13] Polman CH, Reingold SC, Banwell B, Clanet M, Cohen JA, Filippi M (2011). Diagnostic criteria for multiple sclerosis: 2010 revisions to the McDonald criteria. Ann Neurol.

[14] Haller S, Pereira VM, Lalive PH, Chofflon M, Vargas MI, Lovblad KO (2009). Magnetic resonance imaging in multiple sclerosis. Top Magn Reson Imaging.

[15] Willett W (1998). Issues in analysis and presentation of dietary data. Nutr Epidemiol.

[16] Kottek M, Grieser J, Beck C, Rudolf B, Rubel F (2006). World Map of the Köppen-Geiger climate classification update. Meteorologische Zeitschrift.

[17] Mirmiran P, Esfahani FH, Mehrabi Y, Hedayati M, Azizi F (2010). Reliability and relative validity of an FFQ for nutrients in the Tehran lipid and glucosestudy. Public Health Nutr.

[18] USDA Food Composition Databases.

[19] Rosner B, Willett WC (1988). Interval estimates for correlation coefficients corrected for withinperson variation: implications for study design and hypothesis testing. Am J Epidemiol.

[20] Lauer K (2010). Environmental risk factors in multiple sclerosis. Expert Rev Neurother.

[21] Zorzon M, Zivadinov R, Nasuelli D, Dolfini P, Bosco A, Bratina A (2003). Risk factors of multiple sclerosis: a case-control study. Neurol Sci.

[22] Ghadirian P, Jain M, Ducic S, Shatenstein B, Morisset R (1998). Nutritional factors in the aetiology of multiple sclerosis: a case-control study in Montreal, Canada. Int J Epidemiol.

[23] Jahromi SR, Toghae M, Jahromi MJ, Aloosh M (2012). Dietary pattern and risk of multiple sclerosis. Iran J Neurol.

[24] Pekmezovic TD, Tepavcevic DBK, Mesaros ST, Basuroski IBD, Stojsavljevic NS, Drulovic JS (2012). Food and dietary patterns and multiple sclerosis: a case-control study in Belgrade (Serbia). Italian J Public Health.

[25] Riccio P, Rossano R (2015). Nutrition facts in multiple sclerosis. ASN Neuro.

[26] Bagheri M, Maghsoudi Z, Fayazi S, Elahi N, Tabesh H, Majdinasab N (2014). Several food items and multiple sclerosis: A case-control study in Ahvaz (Iran). Iran J Nurs Midwifery Res.

[27] Padler-Karavani V, Yu H, Cao H, Chokhawala H, Karp F, Varki N (2008). Diversity in specificity, abundance, and composition ofanti-Neu5Gc antibodies in normal humans: potential implications for disease. Glycobiology.

[28] Desvergne B, Michalik L, Wahli W (2006). Transcriptional regulation of metabolism. Physiol Rev.

[29] Jump DB, Clarke SD (1999). Regulation of gene expression by dietary fat. Annu Rev Nutr.

[30] Joosen AM, Lecommandeur E, Kuhnle GG, Aspinall SM, Kap L, Rodwell SA (2010). Effect of dietary meat and fish on endogenous nitrosation, inflammation and genotoxicity of faecal water. Mutagenesis.

[31] Shivappa N, Steck SE, Hurley TG, Hussey JR, Hebert JR (2014). Designing and developing a literaturederived, population-based dietary inflammatory index. Public Health Nutr.

[32] Shivappa N, Hebert JR, Behrooz M, Rashidkhani B (2016). Dietary Inflammatory Index and Risk of Multiple Sclerosis in a Case-Control Study from Iran. Neuroepidemiology.

[33] Rego AC, Oliveira CR (2003). Mitochondrial dysfunction and reactive oxygen species inexcitotoxicity and apoptosis: implications for the pathogenesis of neurodegenerative diseases. Neurochem Res.

[34] Albers DS, Beal MF (2000). Mitochondrial dysfunction and oxidative stress in aging and neurodegenerative disease. J Neural Transm Suppl.

[35] Dias V, Junn E, Mouradian MM (2013). The role of oxidative stress in Parkinson’s disease. J Parkinsons Dis.

[36] Zuo L, Shiah A, Roberts WJ, Chien MT, Wagner PD, Hogan MC (2013). Low Po(2) conditions induce reactive oxygen species formation during contractions in single skeletal muscle fibers. Am J Physiol Regul Integr Comp Physiol.

[37] Zhang SM, Hernan MA, Olek MJ, Spiegelman D, Willett WC, Ascherio A (2001). Intakes of carotenoids, vitamin C, and vitamin E and MS risk among two large cohorts of women. Neurology.

[38] Eskandarieh S, Heydarpour P, Elhami SR, Sahraian MA (2017). Prevalence and Incidence of Multiple Sclerosis in Tehran, Iran. Iran J Public Health.

[39] Elhami SR, Mohammad K, Sahraian MA, Eftekhar H (2011). A 20-year incidence trend (1989- 2008) and point prevalence (March 20,2009) of multiple sclerosis in Tehran, Iran: apopulation-based study. Neuroepidemiology.

